# Controlled Construction of Copper Phthalocyanine/α‐Fe_2_O_3_ Ultrathin S‐Scheme Heterojunctions for Efficient Photocatalytic CO_2_ Reduction under Wide Visible‐Light Irradiation

**DOI:** 10.1002/smsc.202100050

**Published:** 2021-07-23

**Authors:** Zhiyuan Mu, Shuangying Chen, Ying Wang, Ziqing Zhang, Zhijun Li, Baifu Xin, Liqiang Jing

**Affiliations:** ^1^ Key Laboratory of Functional Inorganic Materials Chemistry (Ministry of Education) School of Chemistry and Materials Science International Joint Research Center for Catalytic Technology Heilongjiang University Harbin 150080 P. R. China

**Keywords:** CO_2_ reduction, copper phthalocyanine assembly, S-scheme charge transfer, ultrathin α-Fe_2_O_3_, wide visible-light photocatalysis

## Abstract

The wide visible‐light‐driven CO_2_ reduction to acquire solar fuels is a highly desired green route. Herein, novel ultrathin copper phthalocyanine (CuPc)/α‐Fe_2_O_3_ heterojunctions as efficient wide visible‐light‐driven photocatalysts for CO_2_ reduction are controllably synthesized by the hydroxyl‐induced self‐assembly of CuPc onto ultrathin α‐Fe_2_O_3_ as‐pre‐prepared through an Al^3+^‐regulated hydrothermal method. The optimized CuPc/Fe_2_O_3_ heterojunction exhibits about 15‐fold high photoactivity for reducing CO_2_ to CO and CH_4_ compared with reported Fe_2_O_3_ nanoparticles. The exceptional photoactivity is mainly attributed to the enhanced S‐scheme charge transfer and separation in the resulting closely contacted heterojunction, the extended visible‐light range from molecularly disperse CuPc, and its provided central metal cation (Cu^2+^) with favorable catalytic function for CO_2_ activation, mainly by means of the dual‐wavelength photocurrent action spectra, the electrochemical reduction tests, and the in situ diffuse reflectance infrared Fourier transform spectra (DRIFTS). This investigation provides new insight about designing and constructing novel metal phthalocyanine (MPc)‐involved S‐scheme heterojunction photocatalysts.

## Graphical abstract

Novel copper phthalocyanine (CuPc)/α‐Fe_2_O_3_ ultrathin S‐scheme heterojunctions as efficient wide visible‐light‐driven photocatalysts for CO_2_ reduction are controllably synthesized by a hydroxyl inducing assembly process. The optimized CuPc/Fe_2_O_3_ heterojunction exhibits about 15‐fold high photoactivity for reducing CO_2_ to CO and CH_4_ compared with the reported Fe_2_O_3_ nanoparticles.

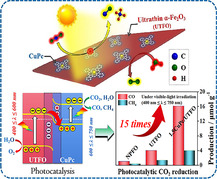

## 1 Introduction

As the principal product of fossil fuel consumption, CO_2_ emissions beyond the recycling capacity of nature has caused the greenhouse effect, which seriously impacts on the climate environment and the development of human society.^[^ ^1^ ^]^ Learning from nature, photocatalytic conversion of CO_2_ into value‐added products by the semiconductors has attracted extensive attention, as a promising eco‐friendly and low‐cost technology.^[^ ^2^, ^3^ ^]^ Hematite (α‐Fe_2_O_3_) as a classic metal–oxide–semiconductor offers significant potential for photocatalysis, owing to its narrow bandgap (2.0–2.2 eV), chemical stability, low‐cost and environmental friendliness, etc.^[^ ^4^, ^5^, ^6^ ^]^ Especially, the excellent oxidation ability of photogenerated holes (h^+^) for water oxidation is an important semi‐reaction during the photocatalytic CO_2_ reduction process as usual.^[^ ^7^ ^]^ However, its low reduction potential of photogenerated electrons (e^−^) deriving from the low conduction band leads to insufficiently competent in CO_2_ reduction reaction by itself.^[^ ^8^ ^]^ Moreover, the short migration distance of holes in α‐Fe_2_O_3_ (less than 4 nm) seriously inhibits the charge separation; meanwhile, unsatisfactory surface catalytic sites should also be overcome.^[^ ^4^, ^9^ ^]^ Therefore, the development of highly efficient α‐Fe_2_O_3_‐based photocatalysts for CO_2_ reduction is full of opportunities and challenges.

In general, to improve the photocatalytic performance of α‐Fe_2_O_3_, morphology control is considered to be one of the most effective strategies.^[^ ^10^, ^11^, ^12^ ^]^ Compared with the common 0D and 1D morphology, the 2D especially for the ultrathin 2D materials (lateral size larger than 100 nm and the thickness typically less than 5 nm) exhibits unprecedented physical, electronic, chemical, and optical properties due to the electron confinement in 2D.^[^ ^13^ ^]^ In prospect of ultrathin 2D α‐Fe_2_O_3_, the photogenerated holes are much easier to migrate to the surfaces, and a larger specific surface area with more catalytic sites is exposed, which are favorable to improve the charge separation and then enhance the photocatalytic activities. Hence, it is meaningful to design a feasible method to synthesize ultrathin α‐Fe_2_O_3_. Gao and co‐workers reported a facile Al^3+^‐intervened hydrothermal method for large‐scaled preparation of cell‐unit‐thick single‐crystalline hematite (α‐Fe_2_O_3_) nanosheets with a thickness of 1.3 nm, and then, they were converted to magnetic maghemite (γ‐Fe_2_O_3_) for utilization in Li‐ion battery.^[^ ^14^ ^]^ Undoubtedly, it is not only necessary but also feasible to study the relationship between thickness and photocatalytic performance. However, few works related to ultrathin α‐Fe_2_O_3_‐based photocatalysts have been reported to date.

In addition, how to overcome the drawback of over‐positive conduction band on α‐Fe_2_O_3_ is still a challenge. In recent years, according to the band structure characteristics of α‐Fe_2_O_3_, construction of Z‐scheme nanocomposite based on α‐Fe_2_O_3_ as the oxidization photocatalyst (OP) with an efficient reduction photocatalyst (RP) has been considered as one of the most effective tactics to overcome the bottleneck of insufficient reduction ability of photogenerated electrons on α‐Fe_2_O_3_ for improving its photocatalytic performance.^[^ ^15^, ^16^, ^17^ ^]^ Wong and co‐workers constructed a hierarchical Z‐scheme α‐Fe_2_O_3_/g‐C_3_N_4_ hybrid with a significantly improved performance for photocatalytic CO_2_ reduction.^[^ ^18^ ^]^ Based on the generally accepted Z‐scheme heterojunction system, Yu and co‐workers proposed a Step‐scheme (S‐scheme) one, and the charge separation at the interface between RP and OP could be further boosted by an internal electric field (IEF) directing from RP to the OP due to the matched Fermi level.^[^ ^19^, ^20^ ^]^ Thus, choosing a proper RP with a matched band structure and Fermi level to construct the ultrathin 2D α‐Fe_2_O_3_‐based S‐scheme heterojunctions as efficient photocatalysts is feasible by forming a strong interaction interface for promoting the charge transfer and separation. In particular, it is highly desired to endow lots of catalytic sites such as nanoclusters or single atoms for CO_2_ reduction.^[^ ^21^ ^]^

Compared with the widely used g‐C_3_N_4_ as the RP, metal phthalocyanine (MPc) as a class of visible‐light response organic photocatalyst is a metal‐central and macrocyclic molecule with a planar conjugated array of 18‐p electrons.^[^ ^22^, ^23^ ^]^ Especially, the high lowest unoccupied molecular orbital (LUMO) and suitable highest occupied molecular orbital (HOMO) energy levels make it possibly used as the RP in the S‐scheme heterojunction photocatalysts.^[^ ^24^ ^]^ Moreover, various central metal ion with M‐N_4_ structures in different MPc could play as a catalytic site, similar to the single atom catalysis.^[^ ^25^ ^]^ It has been demonstrated in our previous work that the zinc phthalocyanine (ZnPc) is used as RP to successfully construct the Z‐scheme nanocomposite with BiVO_4_.^[^ ^26^ ^]^ Remarkably, compared with BiVO_4_ (*E*_g_ ≈ 2.4 eV), α‐Fe_2_O_3_ has a wider spectral response range (*λ* ≤ 600 nm) and a higher matching degree with common MPc materials (550 ≤ *λ* ≤ 750 nm) in terms of solar spectral utilization, which is expected to achieve effective utilization of full visible spectrum. In addition, it is desired to confirm that the positive positions of conduction band and related Fermi level are favored to build a profitable interface driving force by the possible IEF between the α‐Fe_2_O_3_ and MPc. Moreover, it is important to select an MPc with appropriate center metal.^[^ ^27^ ^]^ As various studies have shown that Cu species has a special performance in the catalytic reaction of CO_2_ reduction, it is anticipated that copper phthalocyanine (CuPc) modification would improve the photocatalytic performance of CO_2_ reduction of α‐Fe_2_O_3_ under wide visible‐light irradiation.^[^ ^28^, ^29^ ^]^

In this work, we fabricate a wide visible‐light‐driven S‐scheme CuPc/Fe_2_O_3_ ultrathin heterojunction by a simple hydroxyl inducing assembly process for efficient CO_2_ reduction for the first time. In the assembly process, the pure‐phase ultrathin α‐Fe_2_O_3_ with abundant surface hydroxyl groups induces the highly dispersed CuPc to uniformly assemble on it. Moreover, mechanisms of S‐scheme charge separation and catalytic process are clarified by systematic characterizations. This work provides a vital strategy for the further development of a new type of α‐Fe_2_O_3_‐based S‐scheme heterojunction photocatalysts with excellent performance.

## 2 Results and Discussion

### 2.1 Optimized Synthesis of Ultrathin α‐Fe_2_O_3_

It is meaningful to investigate the effects of additional aluminum ions (Al^3+^) on the properties of as‐prepared α‐Fe_2_O_3_, because Al^3+^ is the crystal face regulator in the synthetic process.^[^ ^14^ ^]^ First, crystal phase compositions of different *x*UTFO UTFO = ultrathin α‐Fe_2_O_3_, *x* = the molar ratio percentage of Al^3+^ to Fe^3+^ in the reactants, and *x* = 0.25, 0.5, and 0.75, respectively), FO (without addition of Al^3+^), and Fe_2_O_3_ nanoparticle (NPFO) samples were characterized by X‐ray diffraction (XRD) patterns. As shown in Figure S1a, Supporting Information, it is noticed that some impurities are formed in FO and 0.75UTFO samples, but 0.25UTFO and 0.5UTFO with pure phase of α‐Fe_2_O_3_ (JCPDS No. 33‐0664) can be obtained in this case. Especially for the 0.5UTFO sample, the relative intensity ratio of the (110) to the (104) facet peak is three times higher than that of the as‐prepared NPFO, because that Al^3+^ ions can be adsorbed on the (001) surface, leading to the preferred growth along [100] direction.^[^ ^14^, ^31^ ^]^ Moreover, a blue shift appears on the absorption edges of 0.5UTFO comparing with other samples via the UV–vis diffuse reflectance spectra (DRS), as shown in Figure S1b, Supporting Information, which is attributed to the quantum size effect.^[^ ^32^ ^]^

Scanning electron microscopy (SEM) images were further used to analyze the morphologies of as‐prepared α‐Fe_2_O_3_ (Figure S2, Supporting Information), and one can see that the resulting FO without additive Al^3+^ is irregular particle shape (Figure S2a, Supporting Information), whereas the 2D nanosheet morphology could be obtained by introducing Al^3+^. Noticeably, the 0.5UTFO (Figure S2c, Supporting Information) shows a thinner and smoother nanosheet structure compared with 0.25UTFO (Figure S2b, Supporting Information) and 0.75UTFO (Figure S2d, Supporting Information), respectively. In addition, the ultrathin nanosheet‐like structure of the 0.5UTFO is further confirmed by transmission electron microscopy (TEM) image (Figure S3a, Supporting Information). The high‐resolution TEM (HRTEM) image shows clear lattice fringes with an interfringe spacing of 0.25 and 0.16 nm (Figure S3b, Supporting Information), matching well with the lattice distance of (110) and (211) planes of hexagonal α‐Fe_2_O_3_, respectively.^[^ ^14^ ^]^ The selected area electron diffraction (SAED) pattern as the inset further reveals that the nanosheets are hexagonal single crystals with (001) orientation, which is consistent with the XRD results.^[^ ^33^ ^]^ Moreover, the atomic force microscopy (AFM) image (Figure S3c, Supporting Information) demonstrates that the thickness of 0.5UTFO is about 1.4 nm, which agrees well with the reported result,^[^ ^14^ ^]^ and as shown in Figure S4a,b, Supporting Information, the 0.25UTFO (≈3.1 nm) is slightly more than twice as thick as 0.5UTFO.

Subsequently, the NPFO as a referential sample was prepared according to our previous reported method, and then, the photocatalytic activities of the as‐prepared *x*UTFO and NPFO samples for CO_2_ reduction were evaluated under visible‐light irradiation (*λ* > 420 nm). As shown in Figure S3d, Supporting Information, 0.5UTFO shows the best activity, which is nearly four times higher than that for as‐prepared NPFO. In the inset of Figure S3d, Supporting Information, the surface photovoltage spectroscopy (SPS) responses confirm that the ultrathin structure of 0.5UTFO is indeed more favorable for charge separation. Moreover, the charge separation related to hydroxyl radical fluorescence spectra (FS) is tested, when the photocatalyst with enough water oxidation capacity is excited in the aqueous environment, and it will produce hydroxyl radicals as captured by the coumarin to produce 7‐hydroxycoumarin, whose fluorescence signal, hence, can be quantified to reflect the charge separation situation of as‐tested sample. In general, a larger signal intensity means a higher photogenerated charge separation. As shown in Figure S5a, Supporting Information, the results of FS are consistent with SPS response. As expected, the 0.5UTFO sample also exhibits the highest photocatalytic activity for phenol degradation under visible‐light irradiation (Figure S5b, Supporting Information). The above‐mentioned results clearly indicate that the controllably synthesized ultrathin α‐Fe_2_O_3_ could exhibit the good charge separation and then the improved photocatalytic performance.

### 2.2 Effects of Modifying CuPc on Ultrathin α‐Fe_2_O_3_

**Figure 1:**
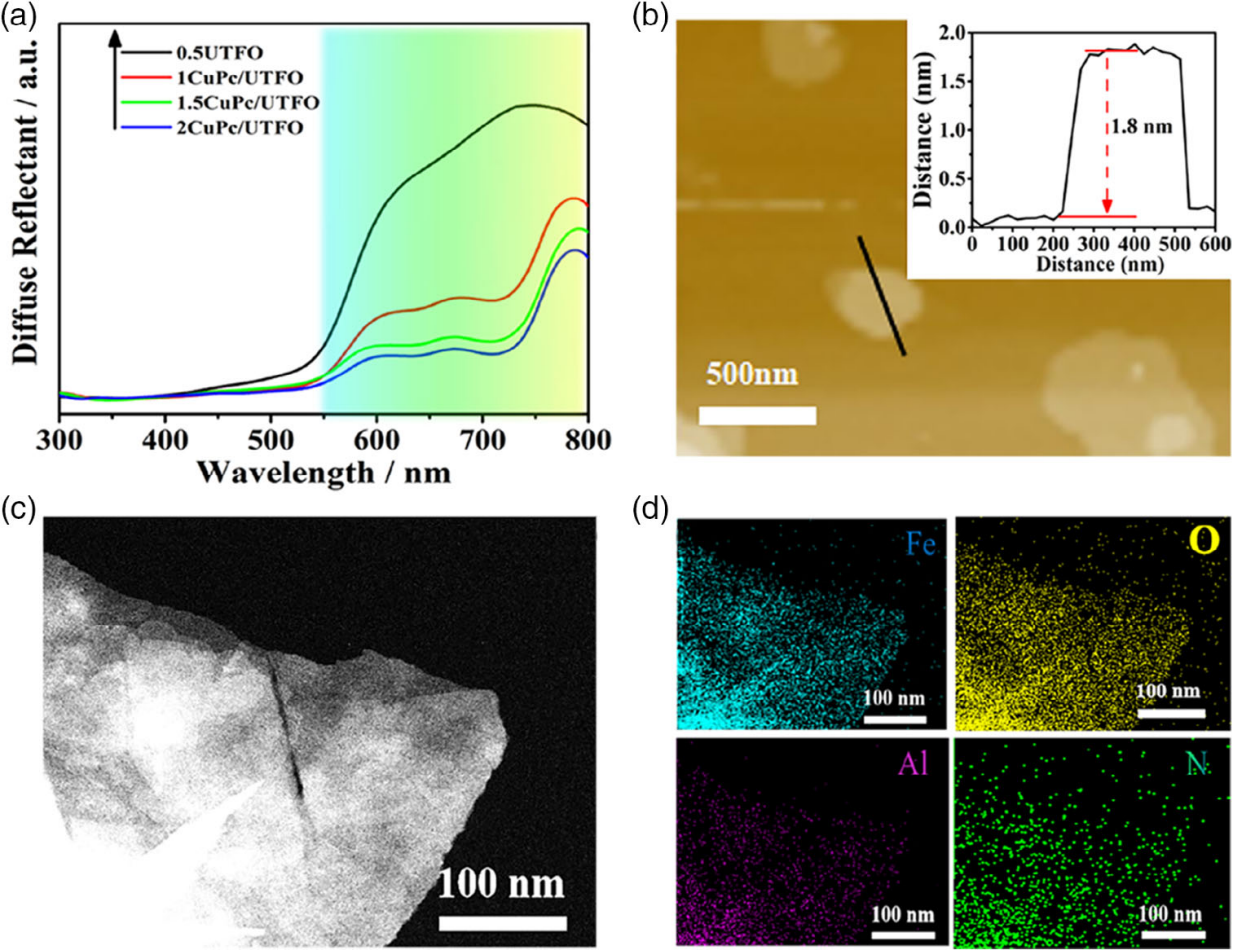
a) UV–vis DRS of 0.5UTFO and *y*CuPc/UTFO, b) AFM image and the corresponding height profile as the inset of 1.5CuPc/UTFO, c) HAADF‐STEM, and d) the corresponding EDX mapping images of elemental Fe, O, Al, and N of 1.5CuPc/UTFO (*y* stands for the mass ratio percentage of CuPc to 0.5UTFO, *y* = 1, 1.5, and 2, respectively).

Based on the XRD patterns of the 0.5UTFO and *y*CuPc/UTFO, as shown in Figure S6, Supporting Information, no detectable characteristic peaks of CuPc are observed, but only pure phase of α‐Fe_2_O_3_ for all samples modified with different contents of CuPc, which maybe attributed to the small amount of CuPc with uniform dispersion on the surface of 0.5UTFO. The DRS of the 0.5UTFO and *y*CuPc/UTFO is shown in **Figure** **1**a, and the results indicate that the introduction of CuPc does not change the absorption band edge of 0.5UTFO, while endows a strong characteristic absorption in the region of 550–750 nm.^[^ ^26^, ^34^ ^]^ As expected, light absorption intensity is increasing with the amount of CuPc. Noticeably, the thickness of 1.5CuPc/UTFO is ≈1.8 nm, which is 0.4 nm thicker than 0.5UTFO according to the AFM images, as shown in Figure 1b, so it suggests that monodispersed CuPc molecules are assembled on the 0.5UTFO. Moreover, the high angle annular dark field scanning transmission microscopy (HAADF‐STEM) (Figure 1c) and the related energy dispersive X‐ray analysis (EDX) mapping (Figure 1d) further confirm that the CuPc is indeed evenly dispersed on the 0.5UTFO ultrathin nanosheets. From the viewpoint of geometric matching (2D/2D), it is accessible that CuPc modifying on UTFO can form an ultrathin heterojunction, and possibly brings with a strong interface interaction.

**Figure 2:**
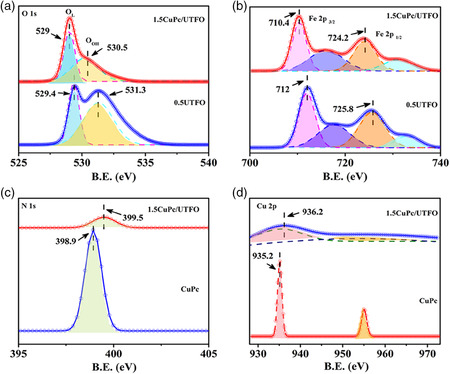
XPS for a) O 1s, b) Fe 2p of 0.5UTFO and 1.5CuPc/UTFO, and for c) N 1s and d) Cu 2p of pure CuPc and 1.5CuPc/UTFO.

To confirm the elemental chemical states and the possible strong interface interaction between the CuPc and the 0.5UTFO, X‐ray photoelectron spectroscopy (XPS) was measured. As shown in **Figure** **2**a, the O 1s spectra of 0.5UTFO and 1.5CuPc/UTFO samples are fitted into two peaks located at 529.5 and 531.6 eV, which are attributed to crystal lattice oxygen and hydroxyl oxygen, respectively.^[^ ^17^ ^]^ Noticeably, the intensity of the hydroxyl oxygen peak at 531.6 eV obviously decreases after be modified by such a small amount of CuPc. As shown in Figure 2b, the binding energy (BE) of Fe 2p element with characteristic two main peaks located at 712.0 and 725.8 eV is attributed to Fe^3+^ in α‐Fe_2_O_3_,^[^ ^35^ ^]^ and the BE has a negative shift after CuPc modification. Meanwhile, the BE of N 2p in the 1.5CuPc/UTFO has a positive shift comparing with pure CuPc (Figure 2c), whereas a slightly positive shift of Cu 2p BE is observed, as shown in Figure 2d. Based on the above‐mentioned XPS analysis, it suggests that a hydroxy group related close interface interaction between the CuPc with 0.5UTFO is formed in the ultrathin heterojunction composite and associated with a process of electron transfer from CuPc to 0.5UTFO in the interface.

**Figure 3:**
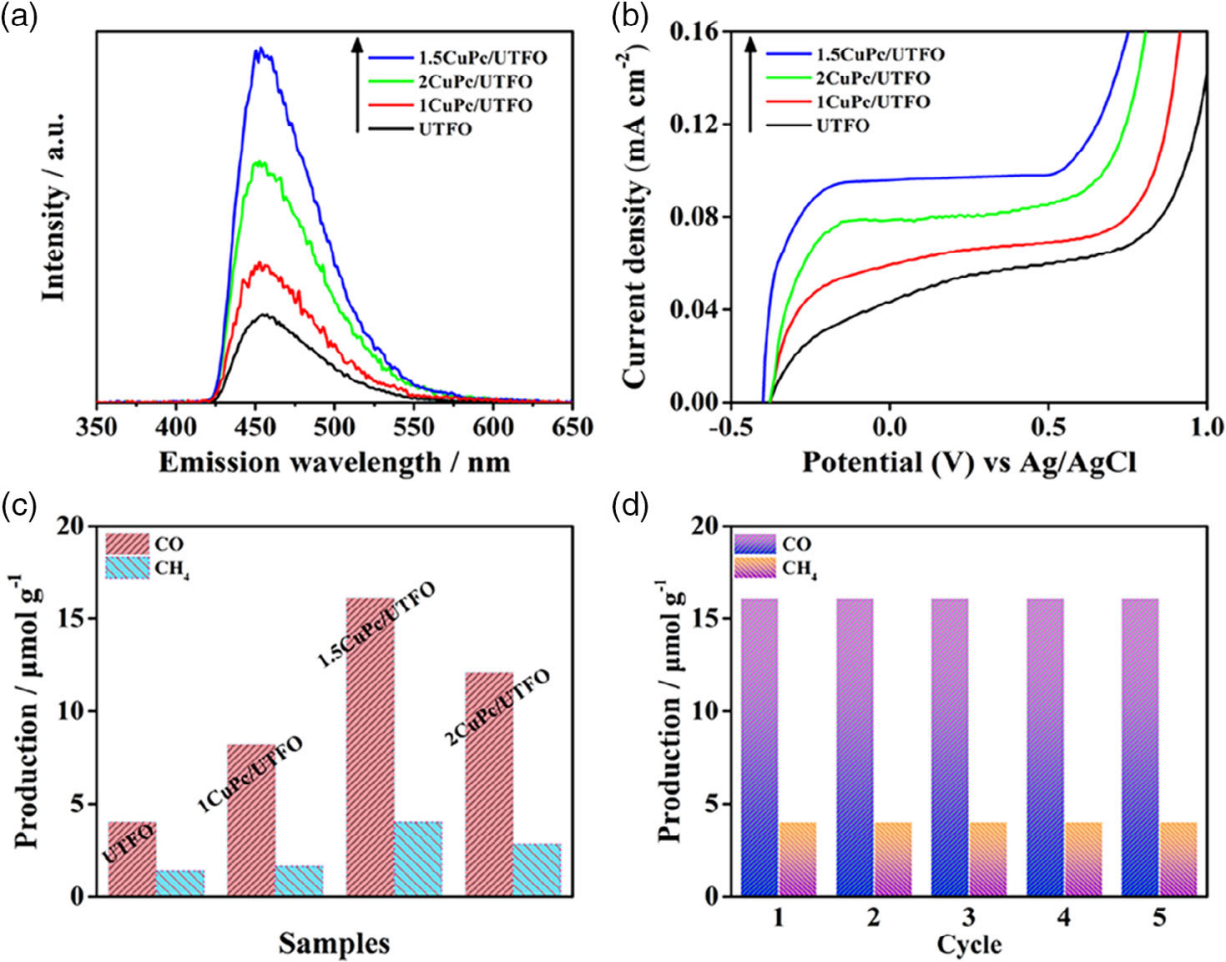
a) Fluorescence spectra related to the formed hydroxyl radicals, b) PEC *I–V* curves, c) photocatalytic activities for CO_2_ conversion under visible light irradiation for 4 h of 0.5UTFO and *y*CuPc/UTFO, and d) photocatalytic cycling test of 1.5CuPc/UTFO.

The effect of CuPc modification on the charge separation of 0.5UTFO is explored by the hydroxyl radical‐related fluorescence spectra (FS). As shown in **Figure** **3**a, the FS signal intensity of 0.5UTFO is significantly increased by introducing an appropriate amount of CuPc, and especially, the one for the 1.5CuPc/UTFO is approximately four times as higher as 0.5UTFO. Moreover, the photoelectrochemical (PEC) measurement further supports the FS results (Figure 3b). The photocatalytic activity of 0.5UTFO is significantly improved after introducing CuPc, and the optimized 1.5CuPc/UTFO heterojunction composite could achieve nearly threefold enhancement (Figure 3c), which is ≈15 times higher than the NPFO; meanwhile, a certain amount of oxidation product O_2_ is also produced, as shown in Figure S7, Supporting Information. Based on the previous results within our group, it is deduced that the generated CO, CH_4_, and O_2_ are all derived from H_2_O and CO_2_.^[^ ^26^ ^]^ In addition, the 1.5CuPc/UTFO sample exhibits good stability for CO_2_ reduction reaction after five cycles (Figure 3d).

### 2.3 Discussion on Mechanism

**Figure 4:**
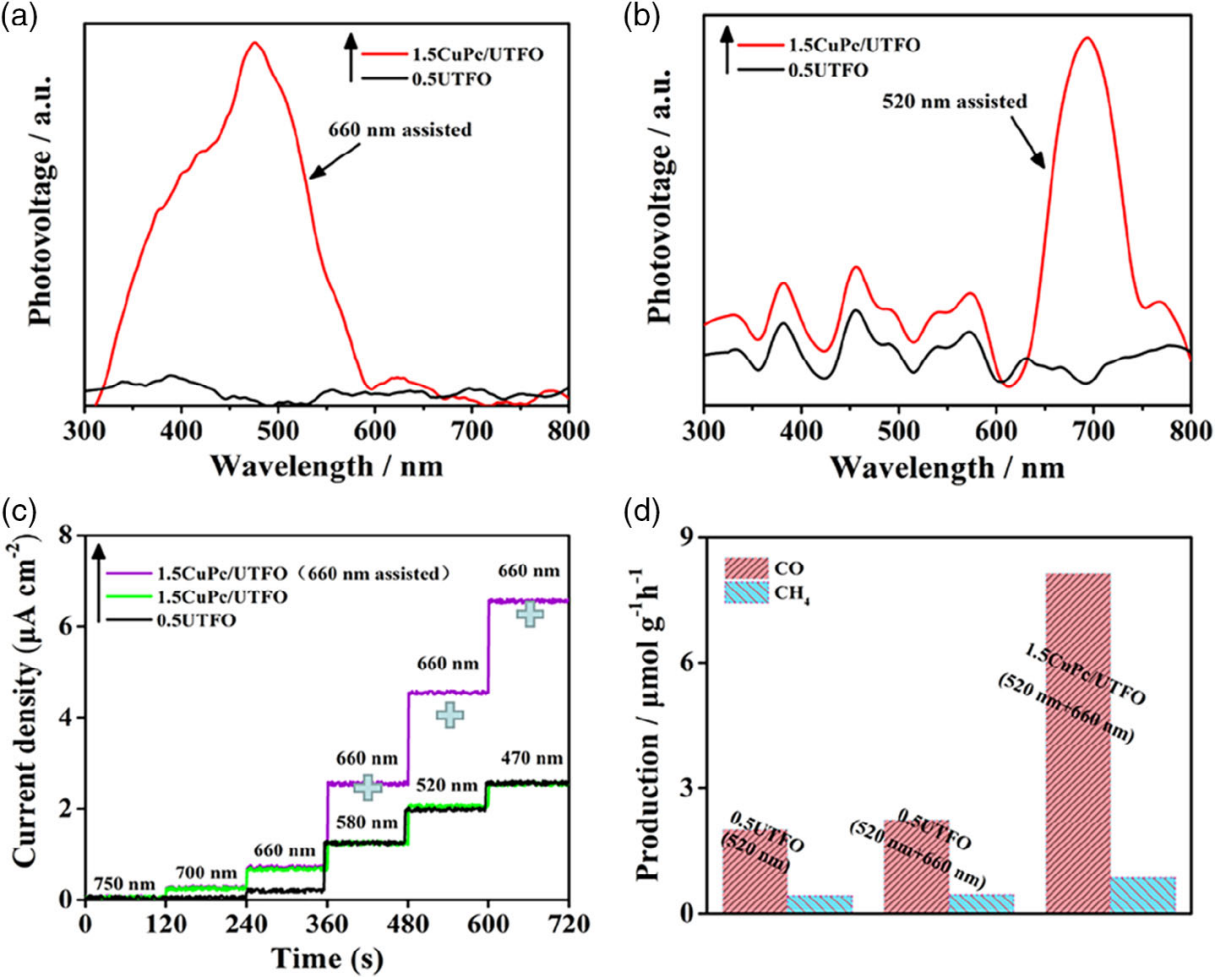
SPS responses of UTFO and 1.5CuPc/UTFO in N_2_ atmosphere assisted with a) 660 nm and b) 520 nm monochromatic light beam. c) Normalized photocurrent action spectra of UTFO and 1.5CuPc/UTFO under different monochromatic light irradiation. d) Photocatalytic activities for CO_2_ conversion under different monochromatic beam irradiation for NPFO, UTFO, and 1.5CuPc/UTFO.

To reveal the mechanism of enhanced charge separation and improved photocatalytic activities on the as‐prepared ultrathin heterojunction composite, the following experiments were designed. According to the energy band alignment of CuPc and UTFO, the S‐scheme charge transfer mechanism is possible. For this, the SPS responses of 0.5UTFO and 1.5CuPc/UTFO are test in a N_2_ atmosphere (**Figure** **4**a,b). Based on the working principle of SPS that when the photocatalyst is excited by the irradiation of light, the photogenerated electrons and holes would reach the surface of material. When the electrons reach the surface and then are captured by the trapping agent (such as oxygen), there is a potential difference on the surface of the photocatalyst, which generates an SPS signal. When nitrogen is used to remove oxygen in the air, SPS signals will not be generated, because surface photogenerated electrons will not be captured. Under this condition, SPS signals can be generated by constructing a channel, which can quickly transfer electrons on the surface of the material, and then, constructing a heterojunction has this function. As shown in Figure 4a, there are no detectable SPS responses for both 0.5UTFO and 1.5CuPc/UTFO, indicating no charge transfer and separation under monochromatic beam probing. However, with an additional 660 nm monochromatic excitation beam, the 1.5CuPc/UTFO exhibits a detectable SPS response in the range of 300–600 nm. Similarly, when the 1.5CuPc/UTFO is exposed to an additional 520 nm monochromatic excitation beam, an SPS response can be detected when probing from 600 to 750 nm. According to the UV–vis DRS spectra (Figure 1a), the light absorption of introduced CuPc is between the range of 550 and 750 nm, whereas 0.5UTFO absorbs light at wavelength smaller than 600 nm. Thus, it is clear that a charge separation‐related SPS response can be detected only when both of CuPc and 0.5UTFO are excited by the excitation and probing beams at the same time, naturally conforming to an S‐scheme charge transfer mechanism.

The proposed S‐scheme mechanism is further confirmed by the monochromatic photocurrent action spectra for the samples of 0.5UTFO and 1.5CuPc/UTFO. As shown in Figure 4c, nearly no photocurrent is detected over the 0.5UTFO under the excitation of monochromatic light with a wavelength longer than 580 nm, but an obvious photocurrent is recorded at 580 nm and increased with the decrease in the excitation wavelength. In construct, obvious photocurrent of 1.5CuPc/UTFO is detected under 700 and 660 nm irradiation, which is mainly attributed to the charge separation from the excitation of CuPc, whereas the photocurrent intensity is nearly equal to 0.5UTFO, as the wavelength continues to decrease, because that only the 0.5UTFO can be excited. Noticeably, the photocurrent density of 1.5CuPc/UTFO is dramatically increased when the wavelength is shorter than 580 nm and with continuous irradiation under additional 660 nm monochromatic excitation beam. These results are in good agreement with the above‐mentioned results of the single‐wavelength‐assisted SPS response. Then, the photocatalytic CO_2_ reduction was performed under single wavelength irradiation and assisted by a single wavelength. As shown in Figure 4d, for the pure 0.5UTFO sample, the activity under 520 nm single wavelength light irradiation is not significantly changed under the condition of simultaneous introduction of 660 nm assisted light, whereas the activity of the 1.5CuPc/UTFO sample under dual‐wavelength light irradiation is significantly improved, which is about four times higher than that of the 0.5UTFO sample. Therefore, its activity result also proves the S‐scheme mechanism of heterojunction composite.

**Figure 5:**
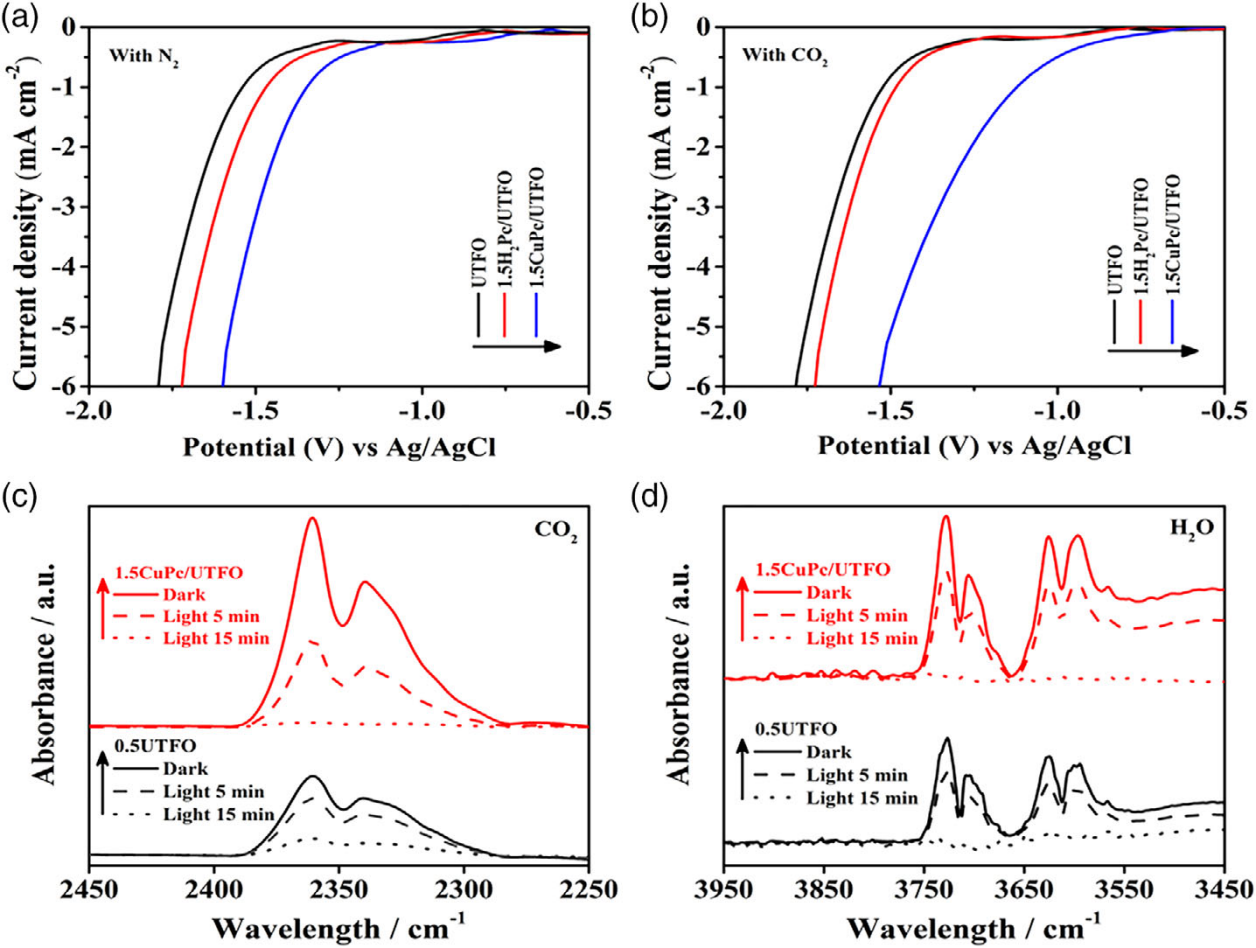
Electrochemical reduction curves of 0.5UTFO, 1.5H_2_Pc/UTFO, and 1.5CuPc/UTFO in a) N_2_‐ and b) CO_2_‐bubbled systems. In situ DRIFTS of adsorbed c) CO_2_ and d) H_2_O on UTFO (black line) and 1.5CuPc/UTFO (red line) (solid line: in dark for 30 min, and dot line: under visible‐light irradiation for 5 and 15 min).

To verify the catalytic function of the coordination metal in CuPc, the electrochemical reduction tests were carried over 0.5UTFO, 1.5H_2_Pc/UTFO, and 1.5CuPc/UTFO samples under the N_2_‐ or CO_2_‐bubbling systems, as shown in **Figure** **5**a,b. The results show that 0.5UTFO and 1.5H_2_Pc/UTFO exhibit similar onset potentials in the N_2_‐ and CO_2_‐bubbling systems, whereas 1.5CuPc/UTFO exhibits a most positive reduction potential, especially in the presence of CO_2_, indicating that the central metal in CuPc has a crucial catalytic effect on the CO_2_ reduction reaction. Moreover, a series of controllable experiments are designed to further confirm the key role of Cu^2+^ in catalyzing the reduction of CO_2_, as shown in Figure S8, Supporting Information. The SPS responses and FS tests are carried on 0.5UTFO, 1.5H_2_Pc/UTFO, and 1.5CuPc/UTFO. The SPS responses and FS spectra both follow the orders: 0.5UTFO<1.5H_2_Pc/UTFO<1.5CuPc/UTFO (Figure S8a,b, Supporting Information), which is in agreement with the photocatalytic CO_2_ reduction activities (Figure S8c, Supporting Information) and phenol degradation activities (Figure S8d, Supporting Information). This indicates that the central metal (Cu^2+^) of CuPc could catalyze the reduction reaction of CO_2_. Furthermore, the CO_2_ and H_2_O adsorption capacities of CuPc as an important factor to photocatalytic CO_2_ conversion were investigated by in situ diffuse reflectance infrared Fourier transform spectra (DRIFTS) at room temperature. As shown in Figure 5c,d, the sample was purged first with CO_2_ gas in advance of passing through H_2_O for 30 min, and then with high purity N_2_ for 1 h. The experimental data of the in situ DRIFTS spectra were obtained by subtracting the background spectrum. As shown in Figure 5c, several vibration bands can be observed in the range of 2280–2390 cm^−1^, which are ascribed to the asymmetric stretching vibration of CO_2_.^[^ ^36^ ^]^ The characteristic peaks of CO_2_ over 1.5CuPc/UTFO are higher than that of over 0.5UTFO, whereas more efficient elimination of the adsorbed CO_2_ over 1.5CuPc/UTFO than 0.5UTFO is observed under visible‐light irradiation for 5 or 15 min, indicating that the introduction of CuPc is favorable for CO_2_ adsorption and followed photocatalytic reduction reaction. Similarly, the peaks in the range of 3550–3750 cm^−1^ are allocated to the surface O—H stretching of H_2_O,^[^ ^37^ ^]^ which are also eliminated more completely over 1.5CuPc/UTFO with more adsorbed water than 0.5UTFO. These results suggest that the excellent CO_2_ reduction performance of 1.5CuPc/UTFO is not only attributed to the good CO_2_ adsorption performance of CuPc, but also owing to the efficient water oxidation process.

**Figure 6:**
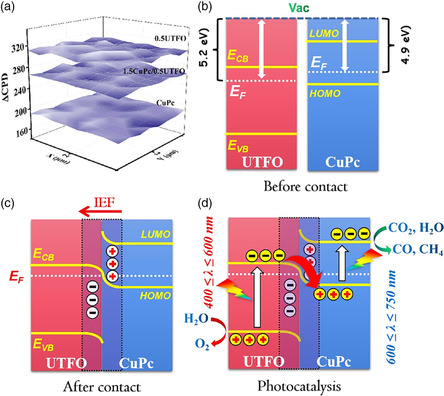
a) SKP maps of 0.5UTFO, CuPc, and CuPc/UTFO. Band diagram and Fermi levels of CuPc and 0.5UTFO b) before and c) after contacting. d) S‐scheme mechanism of photogenerated charge transfer and subsequently induced main reactions in the fabricated CuPc/UTFO heterojunction.

In addition, an effective S‐scheme charge separation between the CuPc and the 0.5UTFO should depend on the strong driving force from IEF. For this, the scanning kelvin probe (SKP) (**Figure** **6**a) and Mott–Schottky plots (Figure S9, Supporting Information) were performed on 0.5UTFO, CuPc, and 1.5CuPc/UTFO, respectively. It is demonstrated that IEF direction from CuPc to 0.5UTFO is formed after close contact due to the different work functions, as shown in Figure 6b,c, which also explains the results of XPS (Figure 2). As a result, IEF is conducive to driving S‐scheme charge transfer in the process of photocatalytic reaction. Finally, the schematic diagram of photocatalytic reaction process is shown in Figure 6d. In this heterojunction system, the CuPc and 0.5UTFO can absorb two completely complementary spectra in the range of whole visible light, and then, the S‐scheme charge separation is driven by IEF, so as to boost the reaction of CO_2_ reduction on CuPc with Cu^2+^ as a catalytic center, whereas H_2_O oxidation occurs on UTFO. In addition, the important role CuPc is also feasible to be replaced with other transitional MPc, such as FePc and CoPc, and the as‐prepared 1.5CuPc/UTFO heterojunction exhibits the best photocatalytic activity for CO_2_ conversion (Figure S10a, Supporting Information), due to the highest charge separation (Figure S10b, Supporting Information) and the best catalytic ability for CO_2_ reduction (Figure S10c,d, Supporting Information).

## 3 Conclusion

In summary, novel ultrathin CuPc/UTFO heterojunction with a Janus structure has been fabricated by a facile hydroxyl‐induced self‐assembly process, which exhibits excellent visible‐light photocatalytic activity for CO_2_ reduction. This is mainly originated from the S‐scheme charge transfer driven by the strong IEF and sufficient utilization of wide visible‐light spectrum. The optimized 1.5CuPc/UTFO exhibits the best activity with 15 times higher than the as‐prepared NPFO, which is attributed to the synergistic effects of the better charge separation and preferable catalytic role of central Cu^2+^ centers of CuPc. In addition, this strategy is applicable to other MPcs for the heterojunction construction. Therefore, this work not only develops a facile synthetic strategy for wide‐spectrum Fe_2_O_3_‐based photocatalysts, but also provides a highly competitive RP for construction of novel S‐scheme heterojunction photocatalysts with wide visible‐light responsive for efficient solar fuel production.

## 4 Experimental Section

4.1

4.1.1

##### Synthesis of Ultrathin Fe_2_O_3_ Nanosheets

The ultrathin α‐Fe_2_O_3_ nanosheets (UTFO) were fabricated based on the previous report.^[^ ^14^ ^]^ Typically, 1 mmol of Fe(NO_3_)_3_·9H_2_O and 0.5 (0, 0.25, or 0.75) mmol of Al_2_(SO_4_)_3_·18H_2_O were dissolved in 10 mL of deionized water under magnetic stirring to form a transparent solution. Subsequently, 3 mL of triethylamine was added into the transparent solution and keep stirring for 40 min. Then, the mixed solution was transferred and sealed in a 50 mL Teflon‐lined autoclave, which was maintained at 160 °C for 24 h. The red precipitates at the bottom of autoclave were collected by centrifugation, washed by water and ethanol for several times, and then dried at 60 °C. After that, the powders were heated at 200 °C for 1 h in muffle furnace. The obtained samples were denoted as *x*UTFO (*x* = the molar ratio of added Al^3+^ to Fe^3+^, *x* = 0.25, 0.5, and 0.75, respectively), and the one without added Al^3+^ was denoted as FO.

##### Synthesis of yCuPc/UTFO Heterojunctions

A series of *y*CuPc/UTFO samples were synthesized using a simple assembly method. Typically, 7.5 mg (5 or 10 mg) of CuPc powder was dissolved into 100 mL dimethyl formamide (DMF) by sonication for 0.5 h and then stirring for 24 h to obtain the CuPc solution. Subsequently, 0.5 g of 0.5UTFO was added into the above‐mentioned CuPc solution; then, the mixture was dispersed in ultrasound for 30 min and keep stirring for 6 h. Finally, the dark red sample was collected by centrifugation, washed by water and ethanol for three times, and then dried at 80 °C for 12 h. The obtained samples were denoted as *y*CuPc/UTFO (*y* = the mass ratio percentage of the added CuPc to 0.5UTFO, *y* = 1, 1.5, and 2, respectively).

##### Synthesis of NPFO

The NPFO was fabricated based on the previous report.^[^ ^30^ ^]^ Typically, 10 mL of 5 wt% ammonia solution was added into a 50 mL Teflon‐lined autoclave, and a 10 mL weighing bottle containing a solution of 0.8 g Fe(NO_3_)_3_ in 8 mL n‐butylalcohol was placed in the autoclave with a support between the autoclave bottom and the weighing bottle. The Teflon‐lined autoclave was kept at 140 °C for 6 h. After being cooled naturally to the room temperature, the sample was washed for several times with ethanol and deionized water, in turn, and then dried in an oven at 80 °C to obtain α‐Fe_2_O_3_. Finally, the samples were calcined in air at 400 °C for 2 h.

##### Characterization Techniques

Powder XRD was performed on a Bruker D8 advance diffractometer with Cu Kα1 radiation (*λ* = 1.5406 Å). UV–vis DRS were recorded on a Shimadzu UV2700 spectrophotometer. Morphologies and element mappings of samples were performed in SEM (ZEISS Sigma 500), TEM (JEOL‐F200) with an energy‐dispersive X‐ray spectrometric microanalysis (EDS) unit, and an AFM (Bruker MultiMode 8). The XPS analysis was recorded on a Kratos‐AXIS ULTRA DLD system with the monochromatic Al Kα X‐rays, and the BE was calibrated internally by the adventitious C 1s peak at 284.4 eV. The steady‐state SPS measurement of the samples was recorded by a home‐built apparatus; the signals were recorded on a phase‐locked amplifier (SRS, SR830) synchronized with a light chopper (SR540), and the monochromatic light was obtained with passing light from a 500 W xenon lamp across a double prism monochromator (SBP300).

In situ DRIFTS were collected from 650 to 4000 cm^−1^ at a spectral resolution of 4 cm^−1^ with 64 scans on a FTIR spectrometer (Nicolet is 50) equipped with a high‐sensitive mercury cadmium telluride (MCT) detector cooled by liquid N_2_ and a DRIFT cell (Harrick) with three windows including an specialized irradiation window. Each sample was first put in a drying oven at 110 °C for 2 h, and then was purged with high purified N_2_ (30 mL min^−1^) at room temperature for 1 h in the in situ tank, to eliminate the physical adsorbed water and other impurities; then, the background spectra were recorded. First, 30 mL min^−1^ pure CO_2_ was introduced into water, and then, the mixture was through the sample surface for 1 h; the spectra, were recorded with subtracting from the background spectrum in dark conditions. Second, the CO_2_ and water were turned off, and 30 mL min^−1^ pure N_2_ was introduced, and the spectra were recorded with subtracting from the background spectrum in simulated sunlight. Thus, the obtained in situ DRIFTS could actually reflect the vibration peaks of the group on the catalyst surfaces in different atmosphere and at different times.

## Conflict of Interest

The authors declare no conflict of interest.

## Data Availability Statement

Research data are not shared.

## Supporting information

Supplementary Material
