# Supplementary material for: Controlled Construction of Copper Phthalocyanine/α‐Fe2O3 Ultrathin S‐Scheme Heterojunctions for Efficient Photocatalytic CO2 Reduction under Wide Visible‐Light Irradiation
Source: Small Sci. 2021 Jul 23;1(10):2100050. doi: 10.1002/smsc.202100050 (PMC11935974; doi:10.1002/smsc.202100050)
Supplement: Supplementary file 1 — Supplementary Material [file SMSC-1-2100050-s001.pdf]

Supporting Information

Controlled construction of Copper phthalocyanine/α-Fe_2O_3 ultrathin S-scheme heterojunctions for efficiently photocatalytic CO_2 reduction under wide visible-light irradiation

Zhiyuan Mu, Shuangying Chen, Ying Wang, Ziqing Zhang, Zhijun Li, Baifu Xin*, and Liqiang Jing**

Table of Contents

1. Experimental Section

1.1 Hydroxyl radicals

1.2 Photoelectrochemical and electrochemical measurements

1.3 Photocatalytic activities for CO_2 conversion

1.4 Evaluation of photocatalytic activity for phenol degradation

2. Supporting Figures

Experimental Section

1.1 Hydroxyl radicals

Hydroxyl radicals (· OH) is an important active species in photocatalytic reaction and related to the charge separation of photocatalysts as usual. The content of hydroxyl radicals can be detected by a fluorescence spectra analysis with coumarin as a labeled molecule. The process was as follows: 0.020 g of the catalyst was added to 20 mL of coumarin solution at a concentration of 1×10^{-3} M. The suspension was stirred for 10 min in the dark to reach the adsorption-desorption equilibrium before the testing. After irradiation for 30 min, appropriate amount of the suspension was collected and removed the photocatalyst by centrifugation, and then transferred into a Pyrex glass cell for the fluorescence measurement of 7-hydroxycoumarin at 332 nm excitation wavelength with an emission wavelength at 460 nm through a fluorescence spectrophotometer (Perkin-Elmer LS55).

1.2 Photoelectrochemical and electrochemical measurements

Photoelectrochemical (PEC) and electrochemical (EC) behavior were measured by a typical three-electrode quartz cell and an electrochemical workstation (IVIUM V13806). The prepared photocatalysts were used as working electrode, a platinum plate (99.9%) was served as the counter electrode, a saturated KCl Ag/AgCl electrode was used as the reference electrode, and 0.5 M Na_2SO_4 solution as the electrolyte. High purity nitrogen gas or oxygen gas (99.999%) was bubbled through the electrolyte before and during the experiment. The illumination source is a 500 W xenon lamp with a cut-off filter (λ> 420 nm). The temperature of each experiment was kept at room temperature (about 25°C).

1.3 Photocatalytic activities for CO_2 conversion

In a typical activities test, a 100 mL stainless steel autoclave with PTFE liner and 3.5 cm^2 area quartz glass window was used as a reactor settling in a circulation cold water bath and keeping the system at 25°C. 0.1g of catalyst was dispersed in 5 mL water, and then high purity CO_2 gas was passed through water bottle and then filled into the reactor with stirring for 20 min. After that, the gas was turned off and continually stirred for another 10 min. A 300 W Xenon lamp was used as the light source with a cut-off filter (λ > 420 nm) followed by irradiation for 4 h. After reaction, 0.25 mL of gas was extracted from the reactor at given time interval for CO and CH_4 concentration analysis by a gas chromatograph (GC-7920, Beijing), and the O_2 concentration was detected with a gas chromatograph (GC-7900, Beijing).

1.4 Evaluation of photocatalytic activity for phenol degradation

0.1g as-prepared sample was putted into a glass reactor with 50 mL phenol solution (10 mg/L) and stirred for 30 min under dark reaching adsorption equilibrium. A 150 W Xenon-lamp was

used as the light source with a cut-off filter (λ > 420 nm) followed by irradiation for 1 h. After reaction, 5 mL suspension was centrifuged and the concentration of phenol measured with a Shimadzu UV-2700 spectrophotometer through the 4-aminoantipyrine spectrophotometric method at the characteristic optical absorption of 510 nm.

Supporting Figures

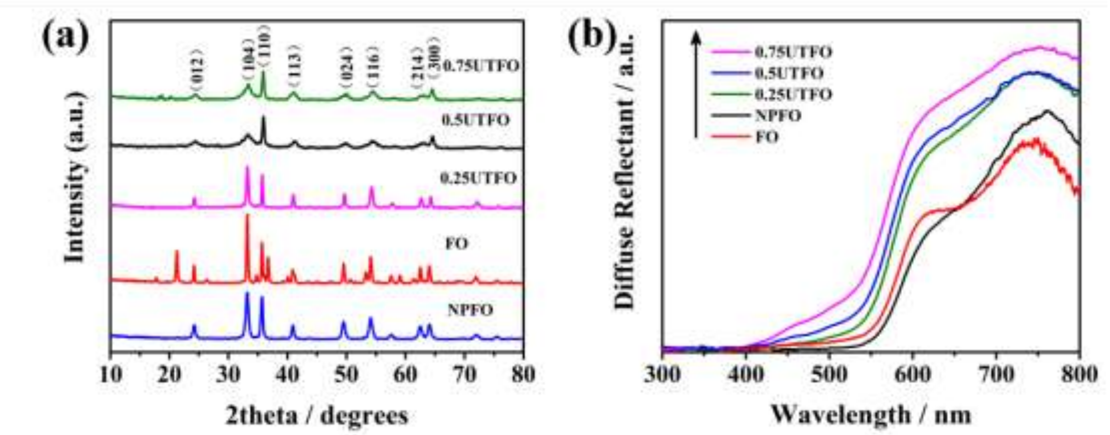

Figure S1. XRD patterns (a) and DRS spectra (b) of NPFO, FO and xUTFO.

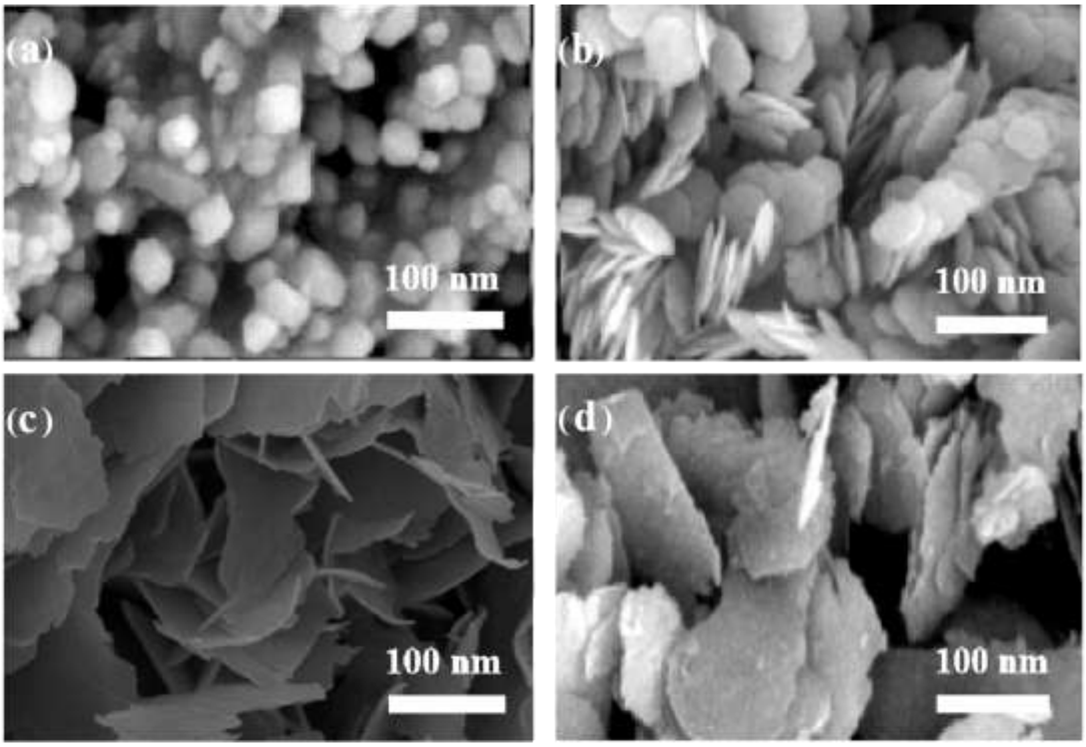

Figure S2. SEM images of FO (a), 0.25UTFO (b), 0.5UTFO (c) and 0.75UTPFO (d).

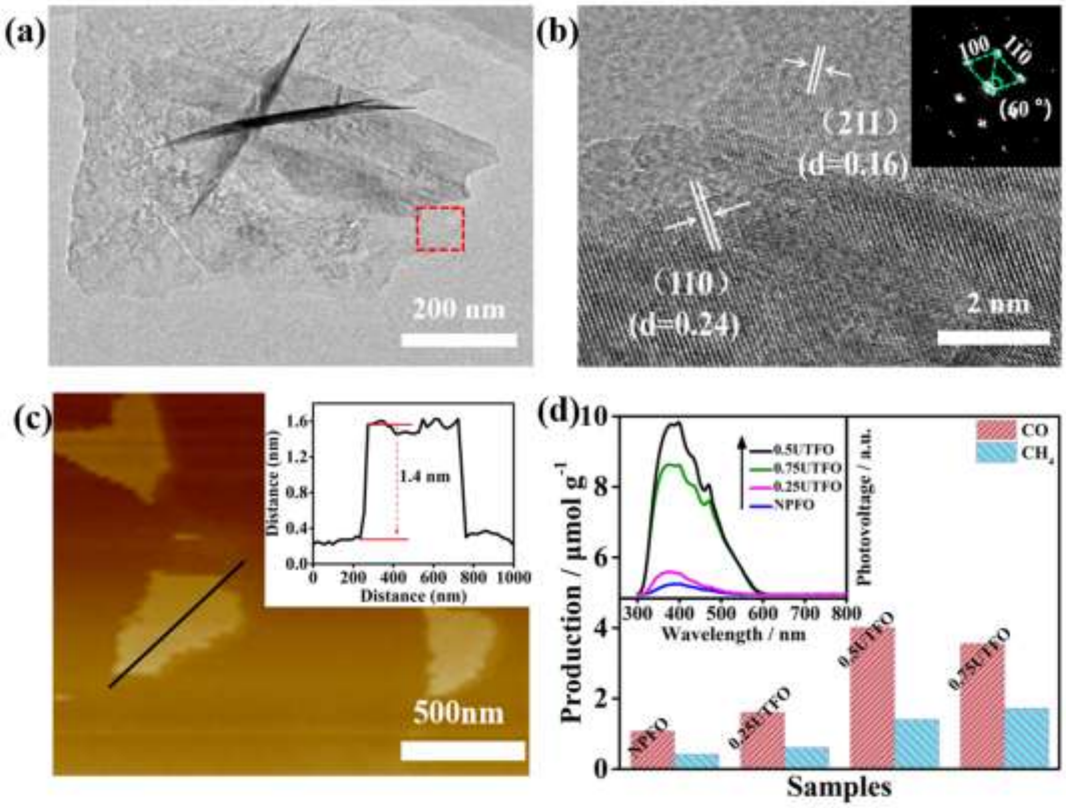

Figure S3. Images of TEM (a) and HRTEM with the SAED pattern as the inset (b), and AFM image with corresponding height profile as the inset (c) of 0.5UTFO. Photocatalytic activities for CO_2 conversion under visible-light irradiation for 4 h and the SPS responses as the inset of NPFO and xUTFO (d). (x stands for the molar ratio of the added Al^{3+} and Fe^{3+} reactants, x= 0.25, 0.5 and 0.75)

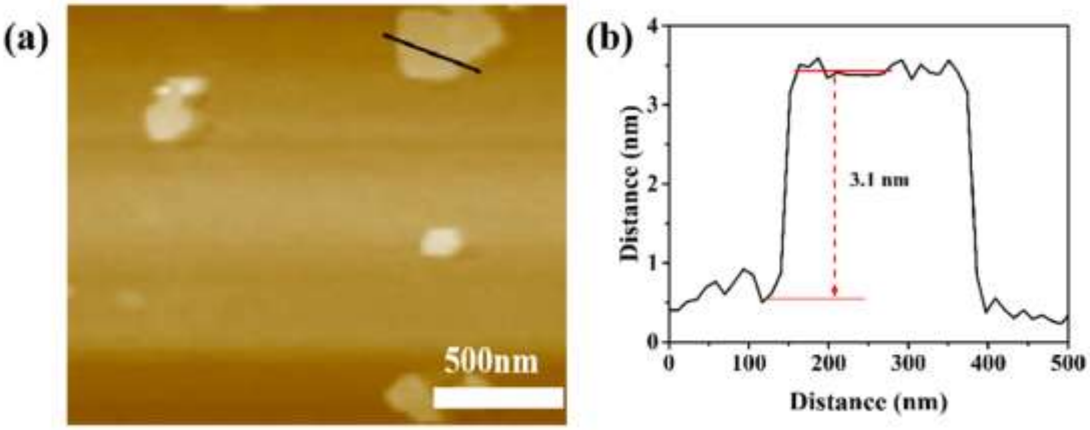

Figure S4. AFM image (a) and the corresponding height profiles (b) of 0.25UTFO.

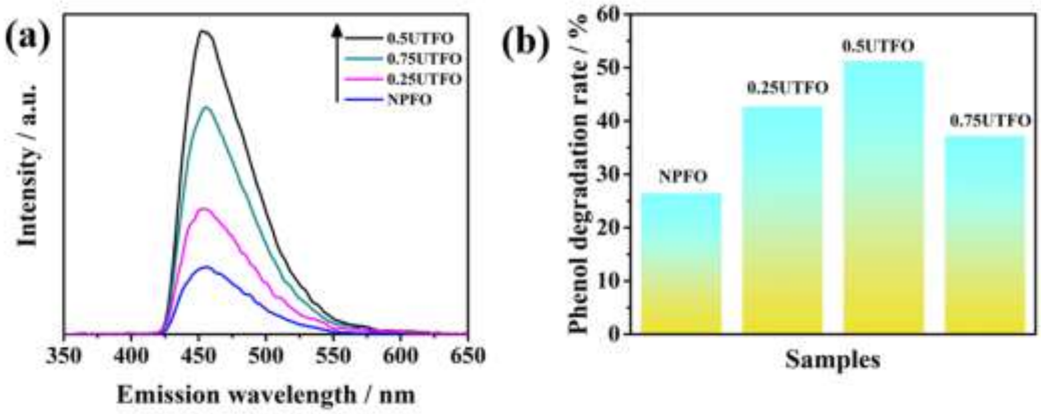

Figure S5. Fluorescence spectra related to the formed hydroxyl radicals (a) and photocatalytic activities for phenol degradation under visible-light irradiation (b) of NPFO and xUTFO.

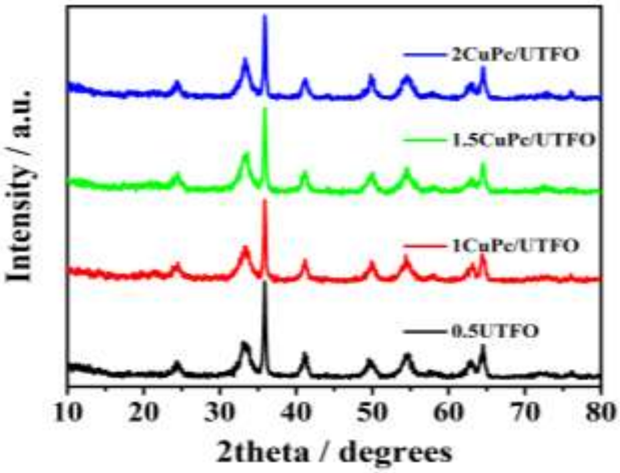

Figure S6. XRD patterns of 0.5UTFO and yCuPc/UTFO

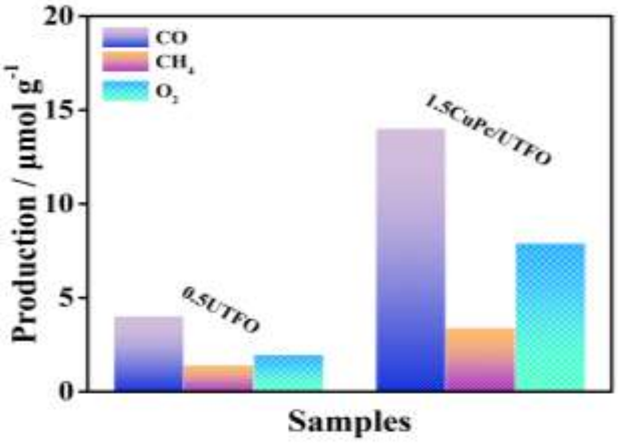

Figure S7. Photocatalytic activities of 0.5UTFO and 1.5CuPc/UTFO for CO_2 conversion under visible-light irradiation.

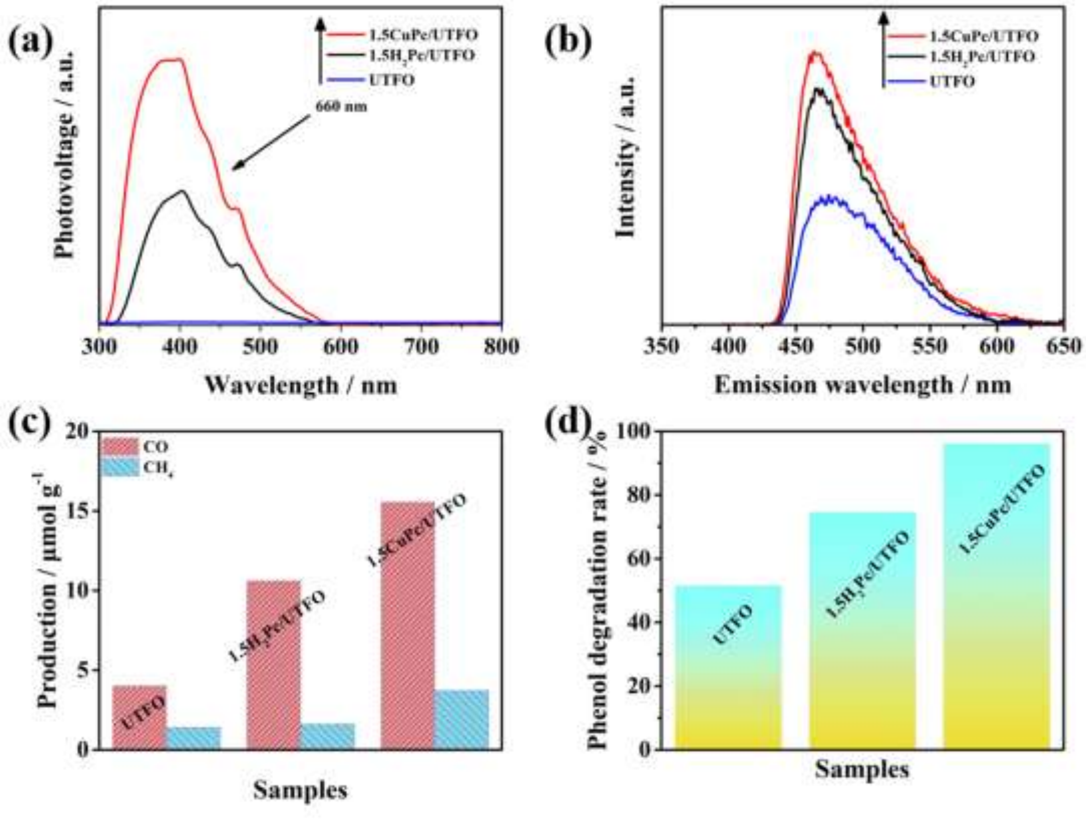

Figure S8. SPS responses assisted with 660 nm monochromatic beam in N_2 atmosphere (a), fluorescence spectra related to the formed hydroxyl radicals (b), photocatalytic activities for CO_2 conversion under visible light irradiation for 4 h (c) and phenol degradation under visible-light irradiation (d) of 0.5UTFO, 1.5H_2Pc/UTFO and 1.5CuPc/UTFO .

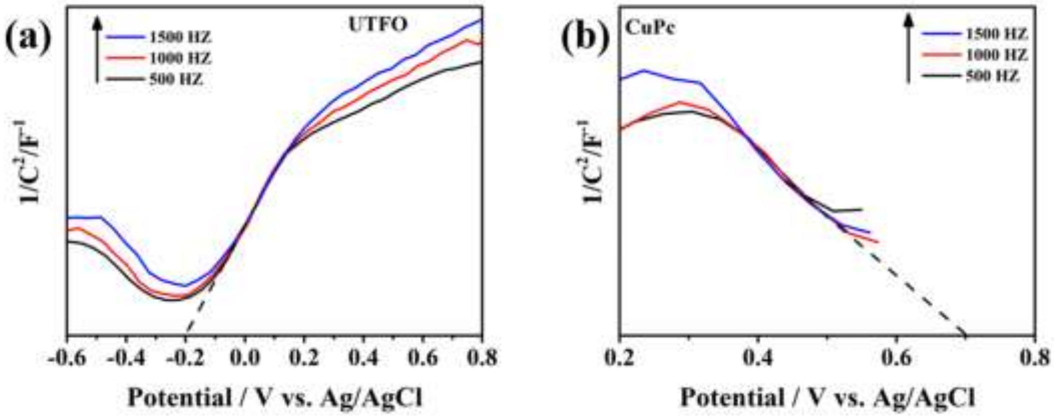

Figure S9. Mott-Schottky plots for 0.5UTFO (a) and CuPc (b).

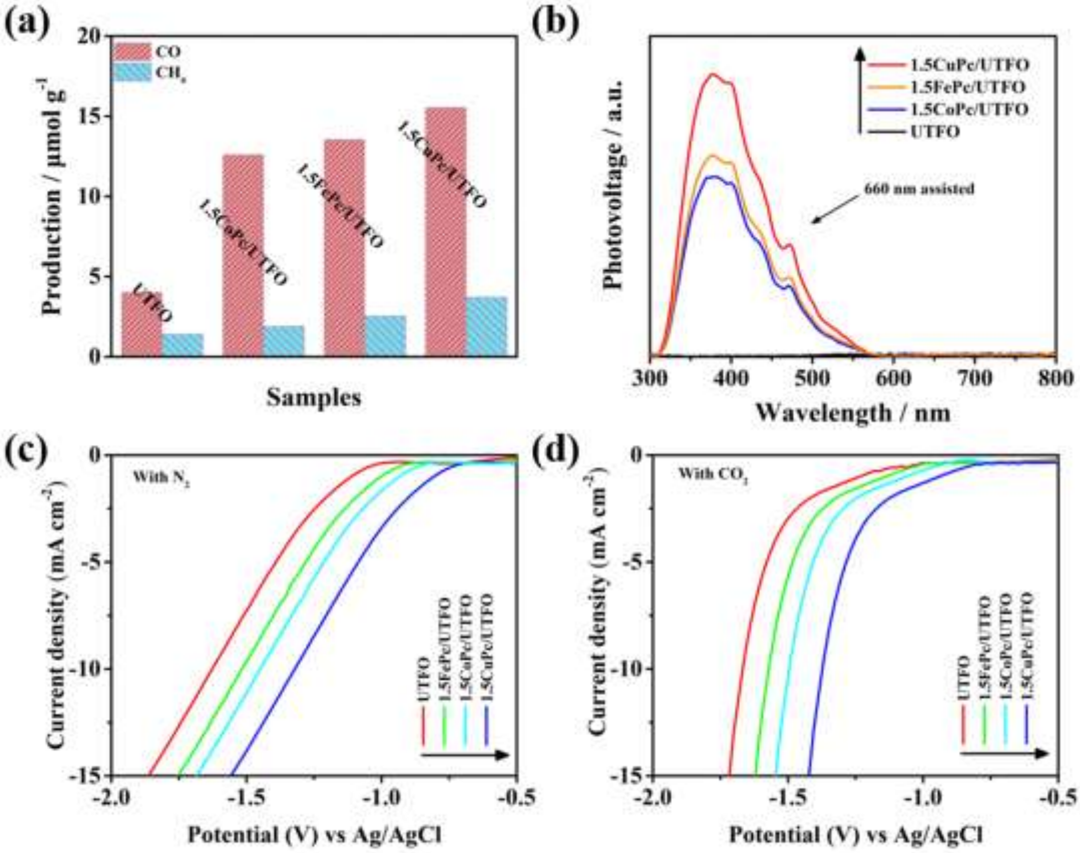

Figure S10. Photocatalytic activities for CO_2 conversion under visible light irradiation for 4 h (a), SPS responses assisted with 660 nm monochromatic beam in N_2 atmosphere (b), electrochemical reduction curves in CO_2 (c) and N_2 (d) bubbled systems of 0.5UTFO and 1.5MPc/UTFO (M= Cu, Fe and Co, respectively).
